# Aspiration-Pneumonia Following Abscess of Steno’s Duct

**Published:** 1896-04

**Authors:** John Greer


### ASPIRATION-PNEUMONIA FOLLOWING ABSCESS OF STENO'S DUCT.[1]

By JOHN GREER, Jr.

While pneumonia in the cow is a common result of the ingestion of foreign and infected particles of food and other material into the respiratory tract, yet the present instance may be of interest, not only as illustrating an unusual origin for such a disease, but as pointing the important moral that early treatment is an essential feature in any specific morbid process in the mouths of the lower animals.

It is a well-recognized fact that there exist normally in the mouth of the lower animals pathogenic bacteria which, under ordinary conditions, produce no evil effects whatever so long as the animal retains a vigorous constitution; so soon, however, as any factors succeed in lowering that vitality the greater is the liability for bacteria to invade the general system and become truly pathogenic for their host. Such, indeed, appears to have been the condition in the case herewith cited.

The subject was a three-year-old cow in which an abscess developed in Steno's duct, the infection doubtless arising through the mouth after the usual manner in which such ascending affections occur. The animal's first symptoms were

[1] Read before the Montreal Veterinary Medical Association, November, 1895.

falling off in the milk, which persisted for four days, when closer examination of the body revealed a small swelling on the left cheek. This rapidly spread downward toward the nostril, and soon infiltrated, likewise, the deeper tissues below the eye on that side; the lachrymal duct became obstructed, and the usual symptoms attending such a condition supervened. Further, there was considerable exophthalmos developed, the eye protruding from the socket to an abnormally great distance, and the conjunctiva was both injected and swollen. After several days the animal refused food, emaciated rapidly, while the swelling gradually increased in size. Hot fomentations were applied with a stimulating liniment, though with little effect. This condition persisted for about three weeks, and, actinomycosis being suspected, some of the pus was removed and examined at the McGill Pathological Laboratory for the fungus, though only with negative result. Soon the secretion of milk had almost ceased, though the condition of the animal remained about the same. At the end of about the third week signs of fever developed, and were accompanied by chills, weakened pulse, and fetid breath, depression and drowsiness, with diarrhœa. These symptoms progressively increased during the next three days, at the end of which time the animal died. This was nearly four weeks after the outset of the local symptoms.

The post-mortem showed a large abscess in Steno's duct, containing about three ounces of yellow, creamy pus, and the surrounding tissues were softened and necrosed. Multiple abscesses were present in the lungs and were surrounded by consolidated areas. Some of the abscesses were of long standing, in which the pus had dried up, and in others the pus was fresher. In all cases the suppuration was connected with the bronchi, which in many places were dilated, and thus produced bronchiectatic cavities. Here then we have a case of aspiration-pneumonia, septic germs being drawn in by inspiration from the abscess in the mouth and setting up secondary abscess in the lungs, and a consequent pneumonia. There were no other signs of septic infection to account for the condition of the lungs, and the cause of death was, therefore, wholly due to this suppurative broncho-pneumonia produced in the way above mentioned.

The case then is of no little importance, as showing the liability to grave pneumonias where an abscess in the mouth remains neglected. The local conditions of the mouth, if long untreated, render the system liable to disease by affecting the constitution, so that a pneumonia can, with much ease, follow the aspiration of the septic germs. I feel sure that had the case been subjected to operative procedure earlier the chances for such infection would have been greatly diminished, and by a proper cleansing of the mouth after operation the animal could have made a fair recovery.

In conclusion I beg to thank Dr. Martin, of McGill Pathological Laboratory, for the kindly interest he has taken in this case.

